# Entropy analysis of stable isotopes in precipitation: tracing the monsoon systems in China

**DOI:** 10.1038/srep30389

**Published:** 2016-08-10

**Authors:** Tao Wang, Jiansheng Chen, Ling Li

**Affiliations:** 1 Geotechnical Research Institute, College of Civil and Transportation Engineering, Hohai University, Nanjing, 210098, China; 2 School of Civil Engineering, The University of Queensland, St. Lucia, QLD 4072, Australia

## Abstract

Due to the complexity of monsoon systems and random behaviors of isotope tracers, conventional methods are not adequate for uncovering detailed information about monsoon activities from typically limited precipitation isotope data. We developed a new approach based on the entropy theory to analyze such data with a focus on the monsoon systems in China, dealing with the complexity of these systems and data deficiency. Using precipitation isotope data from 42 selected stations in and around China within the GNIP network, we computed entropies associated with D and ^18^O. These entropies were found to relate linearly to each other with a proportionality factor close to unity. The spatial variations of the D and ^18^O entropy in the study area revealed the origins, extents and pathways of the Chinese monsoon systems, as well as their interactions. While further investigation is needed at a greater (global) scale, this study has demonstrated how the entropy theory enables an in-depth analysis of precipitation isotope data to trace the pathway and determine the range of a monsoon system.

Stable hydrogen and oxygen isotopes (D and ^18^O) in the water molecule provide a useful tool for meteorological, hydrological, hydro-geological, ecological and agricultural studies^1^,^2^. These isotopes in meteoric precipitation exhibit systematic variations as captured by the Global Meteoric Water Line (GMWL) or Local Meteoric Water Line (LMWL) linked to regional meteorological conditions^3^,^4^,^5^,^6^. A Global Network of Isotopes in Precipitation (GNIP) has been established by the International Atomic Energy Agency^7^ in cooperation with the World Meteorological Organization (WMO) since 1958 to monitor the hydrogen and oxygen isotopes in precipitation worldwide. This network has generated a rich repository of precipitation isotope data, which has contributed to improving our knowledge about global hydrological cycles and climate changes^3^,^4^,^5^,^8^,^9^,^10^,^11^,^12^,^13^,^14^,^15^.

Isotope fractionation occurs during the condensation of water vapor to form meteoric precipitation. This process and the resulting isotope composition of precipitation depend on a number of parameters, including surface air temperature, distance to the coast, altitude, latitude and amount of precipitation^5^. Globally, precipitation isotope compositions have been linked to the atmosphere general circulation predicted by AGC models^8^,^9^,^10^. In China and other Asian monsoon-active regions, isotope variations in the monsoon precipitation are controlled predominantly by the water vapor source and transport pathway. A number of studies have been carried out to explore D and ^18^O as tracers for water vapor source and transport pathway based on the Rayleigh fractionation model in the Asian monsoon regions^11^,^12^,^13^,^14^,^15^,^16^,^17^,^18^. However, these studies focused on localized trends and variations of precipitation isotope in connection with separate monsoon systems.

The two commonly used statistical measures for interpreting the precipitation isotope data are long-term arithmetic mean and long-term weighted mean (weighting according to the amount of precipitation) of δD and δ^18^O^11^,^12^,^13^,^14^,^15^,^16^,^17^,^18^. It should be noted that the IAEA/WMO data, based on monthly composite samples of precipitation, provides only statistically averaged, episodic information about isotopic composition of atmospheric water vapor, resulting from successive precipitation events, each usually with highly variable isotope characteristics^8^. Analysis of precipitation isotope data based on long-term arithmetic mean or long-term weighted mean is not sufficient for revealing fully the links of the isotope with monsoon systems (supplementary information SI1). The composite nature of the sampling and data hinders the development of the isotope method for tracing the water vapor source and transport pathways in regions affected by multiple monsoon systems. Additionally, the analysis suffers from data deficiency due to missing/incomplete data at stations, especially in developing countries^7^,^8^. As a result, a large degree of uncertainty exists with the isotope data analysis – a problem that cannot be resolved directly by the conventional methods based on the Rayleigh fractionation model. This uncertainty problem is further escalated due to the complexity of the monsoon system. For example, the East Asian monsoon system is complicated by various factors including ElNiño Southern Oscillation (ENSO), surface sea temperature, Eurasian snow cover, downward propagation of stratospheric anomalies associated with the polar vortex, and atmospheric internal processes such as Arctic Oscillation (AO), North Atlantic Oscillation and planetary wave activities^19^,^20^,^21^.

The uncertainty problem with the isotope data analysis may be alleviated by the entropy theory, which is ideal for dealing with stochastic systems and insufficient data. Entropy, as a measure of the degree of dispersion, uncertainty, disorder and diversification of a system, can better reflect the complexity of monsoon systems^22^. The principle of maximum entropy enables the extraction of as much information as possible from data with as little assumption as possible made^23^,^24^,^25^. Within a monsoon system, its entropy would continuously increase along the vapor travel path. The rate of entropy increase, however, intensifies when mixing between the original vapor and locally generated vapor takes place. The trend of entropy increase would break where two monsoon systems travelling in the opposite directions meet. An analysis of the precipitation isotope data based on these entropy behaviors would help to trace the monsoon systems. Typically an entropy-based method is efficient, requiring relatively little computational effort, and is versatile in its applicability across many disciplines, such as ecology, biology, economics, hydrology and water resources^26^,^27^,^28^,^29^. Here we aim to develop such a method for analyzing precipitation isotope data to study the monsoon systems in China, dealing with the complexity of these systems and data deficiency.

China occupies most part of East Asia with an area of 9.6 million square kilometers and terrains of three ladders in altitude from the west to east^30^,^31^. The climate in China is controlled by two seasonal monsoons, namely, the winter monsoon and summer monsoons consisting of Indian monsoon and East Asian monsoon^32^,^33^ (Fig. 1). The winter monsoon is a flow of cold, dry air associated with the Siberian-Mongolian High and the westerlies, which has a great influence on the precipitation in northern China. In contrast, the summer monsoons are steady flows of warm, moist air from the tropical Indian and Pacific oceans, transporting water vapor to southern China^34^. The winter monsoon and summer monsoons form three corridors of vapor inflow to China: northwestern corridor, southwestern corridor and southeastern corridor, with vapor sourced mainly from inland, the Indian Ocean and the Pacific Ocean, respectively^35^.

The onset, extent, peak and retreat of monsoon seasons in China vary with the relative intensities of these three monsoon streams^32^,^33^. In the summer, the Indian monsoon and East Asian monsoon have larger intensities than that of the northwestern monsoon, while the opposite occurs in the winter. The onset of the monsoon season begins with heavy rainfalls over South China Sea in mid-May. Subsequently the rain belt gradually moves to the north and northwest from coastal seas toward inland areas. The peak rainy season tends to occur in late June over the Yangtze River region and in late July over northern China. As the northwestern monsoon strengthens against the Indian monsoon and East Asian monsoon after July, the summer monsoons withdraw progressively from northern China southward toward coastal regions. On a yearly cycle, the summer monsoons bring a vast amount of rainfall to China, due to the warm vapor and strong intensity of the monsoons compared with the winter monsoon of cold continental air mass^35^,^36^,^37^. In southern China, the Indian monsoon is the main contributor of water vapor, with an average intensity about two times that through the southeastern corridor^35^. However, in central and northeastern China, the East Asian monsoon is the main source of the precipitation^38^.

As mentioned above, our understanding of monsoons traced by precipitation isotope in China is far from being complete. This paper aims to develop and apply a new method based on the entropy theory to examine the random behavior of precipitation isotopes and explore the monsoon systems in China using long-term isotope data from 42 selected GNIP stations. This is the first time that the entropy theory is applied to interpret the precipitation isotope data.

## Results

### Relation of D and ^18^O entropies

The relation between D and ^18^O concentrations in natural meteoric water has been determined as *δD* = *8δ*^*18*^*O* + *10* (Eqn. 1), which has been applied as a standard method for examining the D and ^18^O variations in natural waters^3^,^4^. Based on the precipitation isotope data collected from the 42 selected GNIP stations, we computed the entropies of D and ^18^O (details in the Method section). Another linear relationship was found between these two isotopes in terms of entropy (Fig. 2). Linear regression of the calculated D and ^18^O entropies yields *ED* = (*0.93* *±* *0.05*)*E*^*18*^*O* + (*0.98* ± *0.07*) (Eqn. 2) with a high correlation coefficient of *R*^2^ = 0.91, where ED is the entropy of D and E^18^O is the entropy of ^18^O. The maximum and minimum values for ED are 2.72 and 1.71, and for E^18^O are 1.79 and 0.89, respectively.

The isotope composition of meteoric water characterized by Eqn. 1 reveals different degrees of fractionation between D and ^18^O due to their different mass numbers. The entropies of D and ^18^O reflect the same/similar degree of dispersion and uncertainty of monthly averaged D and ^18^O values in precipitation. Therefore, the difference between the behaviors of these two entropies, if any, is expected to be small, as shown by their linear relationship with the proportionality factor close to unity (Eqn. 2).

### Link between spatial entropy variations and monsoon systems

In the Asian monsoon-active area, the vapor source and monsoon pathway, as mentioned above, are the main factors that determine the precipitation isotope distribution^11^,^12^,^13^,^14^,^15^,^16^,^17^,^18^. Changes in the variance of the precipitation isotope distribution lead to variations of isotope entropy. Therefore, the variations of the D and ^18^O entropy could be used to infer the vapor source and monsoon pathway as discussed in detail below. On the other hand, we have tested and found no relation of the D and ^18^O entropy with the annual average precipitation or annual average temperature, indicating negligible effect of both factors on the variations of the precipitation isotope entropy (supplementary information SI2).

Figures 3 and 4 present the D and ^18^O isoentropy lines in the study area, respectively. The patterns shown in both figures share a large degree of similarity and portray well the links of the D and ^18^O entropy with vapors sourced from different monsoon streams: the winter monsoon with four streams (W1-4), the summer Indian monsoon with two streams (I1-2) and the summer East Asian monsoon with three streams (E1-3). These monsoon streams are drawn along the paths with minimum entropy gradients (i.e., least rates of entropy increase). Overall the isotope entropies associated with the winter monsoon are higher than those in the summer monsoons. The maximum ED and E^18^O in the winter monsoon are 2.6 bit and 1.7 bit, respectively. These values are larger than those in the summer monsoons with max(ED) = 2.3 bit and max(E^18^O) = 1.4 bit for the Indian monsoon, and max(ED) = 2.1 bit and max(E^18^O) = 1.2 bit for the East Asian monsoon. The differences are likely to be caused by different vapor origins. While the vapor in the winter monsoon is sourced from the secondary evaporation from inland water, the summer monsoon acquires vapor from the warm ocean. Where the Indian monsoon crosses the southwestern border of China, the isotope entropy values are larger than those where the East Asian monsoon crosses the southeastern border of China. This may be due to a longer journey taken by the water vapor from the Indian Ocean to the Indo-Chinese border.

The results also show a general trend of decreasing entropies of D and ^18^O from the northwest to southeast, reflecting the combined influence of different monsoons. The isoentropy lines in the northwest and southeast, where a single monsoon system (winter or summer East Asian monsoon) dominated, are sparse, showing relatively small spatial variations. In contrast, the isoentropy lines are relatively dense with large gradients in central China and the south-central part of northeastern China, where the summer monsoon and winter monsoon meet. The dense isoentropy lines indicate a great impact on the entropy of precipitation isotope, imposed by the interaction between two monsoon systems.

If a monsoon system is assumed to be isolated, then its entropy should continuously increase along the travel path based on the principle of maximum entropy. If the entropy of a monsoon system decreases, it would have been influenced by another system, which brings negentropy via vapor mixing. Figures 3 and 4 reveal that the precipitation isotope entropies of the Indian monsoon and winter monsoon decrease along the path from the border: from 2.3 bit to 2.1 bit for the D entropy and from 1.4 bit to 1.1 bit for the ^18^O entropy in the Indian monsoon, and from 2.6 bit to 2.4 bit for the D entropy and from 1.7 bit to 1.5 bit for the ^18^O entropy in the winter monsoon. The entropy reduction in the winter monsoon is smaller than that in the Indian monsoon. This is likely to be due to smaller rainfall events involved with the winter monsoon than those with the Indian monsoon. In contrast, the journey of the East Asian monsoon, mostly over the Pacific Ocean, leads to increase in both isotope entropies along the path, indicating less interaction with external systems.

### Tracing monsoon systems

Given that the spatial variations of the D and ^18^O entropy are largely controlled by the vapor source and monsoon streams, we suggest that the D and ^18^O entropy in precipitation can be applied to trace monsoon systems. The patterns shown in Figs 3 and 4 indicate that one stream of the Indian monsoon (I1) passes eastward from the southwestern border of China and then turns to the northeast of the south central China before reaching eastern China, where its intensity weakens after producing heavy rainfalls. Overall the Indian monsoon transports large water vapor from the Indian Ocean to China. Note that a fraction of the Indian monsoon (I2) turns to the north at the eastern Qinghai-Tibet Plateau, which may result from the impeding effect of the Hengduan mountain^37^. There is a similar case, mirrored by the parallel isoentropy lines along the Himalayas (Figs 3 and 4), that the Himalayas impede the movement of the Indian monsoon from the south to north and the movement of the winter monsoon from the north to south.

After crossing the northwestern border, the winter monsoon commences its journey in a stream (W1) from the northwest to the south until reaching the Qinghai-Tibet Plateau; and another stream (W2) moves to the east until reaching the central part of northern China. The third stream of the winter monsoon (W3) from the north brings some precipitation to the north central China. The fourth stream of the winter monsoon (W4) from the north affects the precipitation in the north part of northeastern China.

Across the southern and eastern coastal areas of China, the isoentropy lines vary a little. This is likely to be due to the influence of the East Asian monsoon stream (E1) bringing water vapor from the Pacific Ocean^36^. Another two streams of the East Asian monsoon, E2 and E3, travel northwestward from the Pacific Ocean, and then mix with the winter monsoon in central China and the south-central part of northeastern China, bringing water vapor and producing most of the precipitation in these areas.

## Discussion and Concluding Remarks

The method introduced in this study on the basis of the entropy theory has enabled an in-depth analysis of precipitation isotope data to explore complex monsoon systems. Compared with previous studies based on meteorological data, the present analysis uses precipitation isotope data with longer time series and wider spatial coverages. The results given by this method are based on the principle of maximum entropy, and thus represent the most probable course of events. The travel paths of the summer and winter monsoons and their influences discussed above are generally consistent with findings of previous studies^12^,^13^,^14^,^15^,^16^,^17^,^18^.

The study has assumed that the precipitation isotope follows the normal distribution. We have tested this assumption and found that the normal distribution fits reasonably well with the isotope data. While future investigation can be carried out to examine further the statistical distribution of precipitation isotope, we believe the main conclusions from this study hold, in particular, the usefulness of the entropy method for studying the monsoon systems. The linear relationship between the D and ^18^O entropy requires further investigation. The question whether it is a generic relation similar to the GMWL should be addressed. It should be pointed out that there is an anomalous data point in Fig. 2 (point 2 for Changchun). To examine this, we calculated the differences between the calculated entropy values and those given by the fitted equation for all stations (Table 1). The results show no correlation with longitude, latitude, precipitation, temperature or monsoon pathway. These differences do not follow the normal distribution either (supplementary information SI3). What causes the deviation needs to be further studied. As the precipitation isotope data continue to accumulate, the new method can be adapted to explore the seasonality of the monsoon systems based on monthly or even daily averaged data.

## Methods

### Entropy theory

Entropy has been widely applied as a measure of dispersion, uncertainty, disorder and diversification associated with a random variable and its probability distribution, since the development of the information entropy theory in the late 1940s^22^. The concept is derived from the second law of thermodynamics with a few modifications. In generalized thermodynamics, entropy^26^,^27^ is decomposed into two parts *dH* = *dH*_*e*_ + *dH*_*i*_, where *dH*_*e*_ is the entropy exchange between the system and its surroundings and *dH*_*i*_ is the entropy produced in the system itself. In an isolated system, the entropy tends to increase to reach the maximum value – a chaotic state of the system. That is to say that *dH*_*i*_ will be increasing until the system comes under the condition of dynamic equilibrium. In contrast, an open system undergoes continuous exchange of material, energy and information with the ambient environment, which brings negentropy to reduce the entropy of the system and hence helps to maintain its order^23^.

### Principle of maximum entropy

This principle introduced by Jaynes^23^,^24^,^25^ states that the entropy of a closed system increases to reach a peak value. It has been extensively used in non-thermodynamic fields. The process of problem-solving could be regarded as extracting information from the provided data, including known data and assumptions to unknown information. When the data are insufficient, the solution with the maximum entropy must correspond with the provided data under the condition of the least amount of assumptions about the unknown information. In other words, the principle of maximum entropy refers to the case in which as much information as possible can be obtained based on as few assumptions as possible. The case also means the most probable course of events, not the only possible ones. An optimal solution should be the one with the maximum entropy among all the solutions, subject to specified constraints.

### Entropy calculation

The entropy equation for a random variable is given by *H* (*X*) = −k∫*p* (*x*) log*p* (*x*)*dx*, where *p*(*x*) is the probability density function. The units of Binary Digit (bit), Natural Digit (nat) and Decimal Digit (dit) are used with the base of the logarithm of 2, e and 10, respectively^22^. In most cases, the base of the logarithm of 2 is adopted in the calculation with *k* = 1. Entropy equations for random variables following the normal distribution and other distributions such as the gamma distribution, lognormal distribution, beta distribution and extreme distribution have been derived based on the principle of maximum entropy^39^. The general equation for computing entropy based on the normal distribution is given by 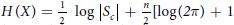 (Eqn. 3), where *n* is the set number of variables and *S*_*c*_ is the cross correlation matrix of all sets of random variables^39^,^40^,^41^,^42^. When Eqn. 3 is used for a single variable instead of multivariate data, *n* is equal to 1 and *S*_*c*_ becomes the variance of the random variable. It has been found that precipitation data follow the normal distribution^40^,^41^,^42^. We tested and showed that the precipitation isotope data also follow reasonably well the normal distribution. Thus Eqn. 3 was used in calculating the isotope entropy.

### Data used in the analysis

**Figure 1:**
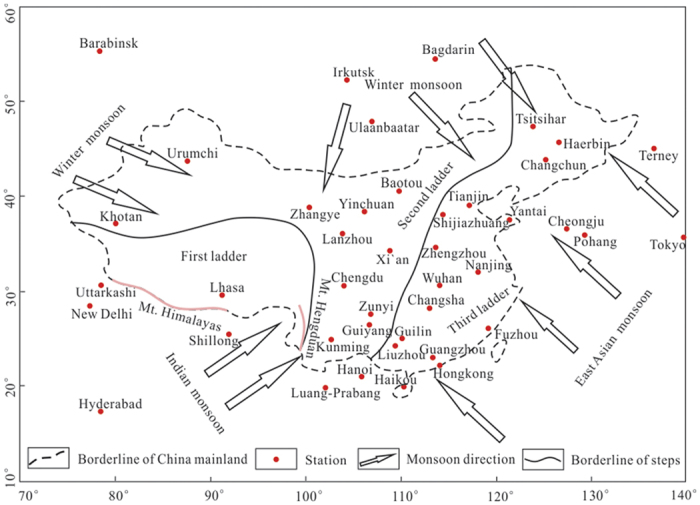
Location map of China and selected stations as well as the three monsoon systems, modified from reference^6^ using CorelDRAW X6 (**http://down.52pk.com/xiazai/13513.shtml**). Mt. means mountain. The pink solid lines show the Himalayas and Hengduan Mountains. The arrows indicate the directions of the winter monsoon, Indian monsoon and East Asian monsoon.

**Figure 2:**
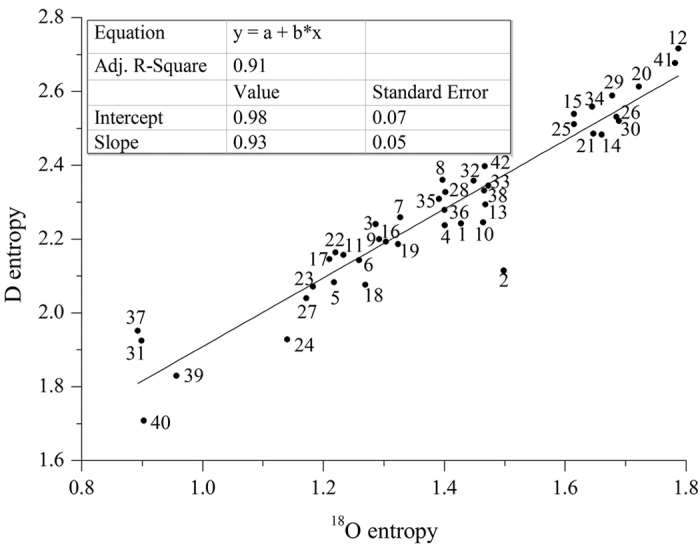
Relation between the D entropy and ^18^O entropy. The station number is listed in Table 1.

**Figure 3:**
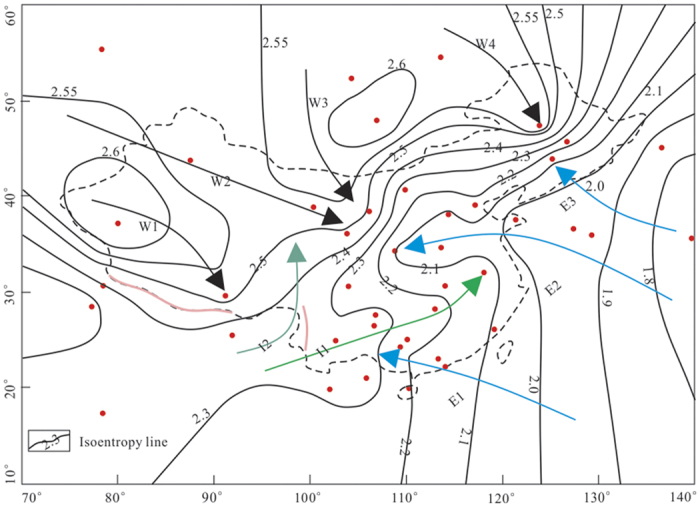
D isoentropy lines in the study area, generated using Surfer^®^ [12] from Golden Software, LLC (**www.goldensoftware.com****) and overlaid with** Fig. 1 **with a few modification using CorelDRAW X6 (****http://down.52pk.com/xiazai/13513.shtml**). W1, W2, W3 and W4 with black solid arrows indicate the pathways of the four streams of the winter monsoon; I1 with green solid arrow and I2 with light green solid arrow indicate the pathways of the two streams of the Indian monsoon; and E1, E2 and E3 with blue solid arrows indicate the pathways of the three streams of the East Asian monsoon.

**Figure 4:**
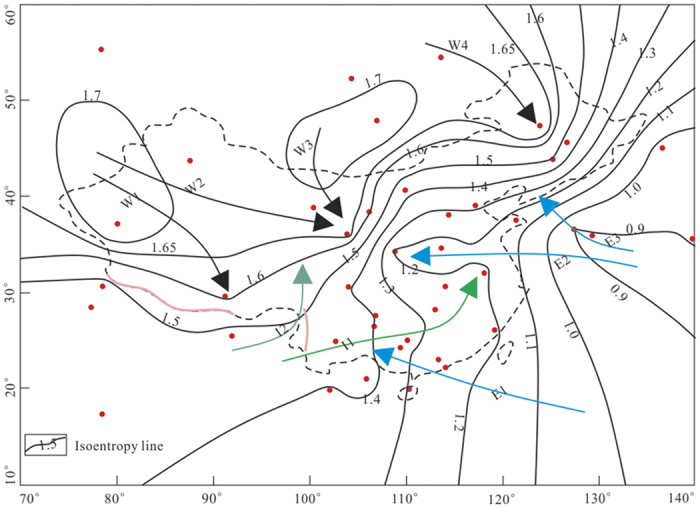
^18^O isoentropy lines in the study area, generated using Surfer^®^ [12] from Golden Software, LLC (www.goldensoftware.com) and overlaid with Fig. 1 with a few modification using CorelDRAW X6 (http://down.52pk.com/xiazai/13513.shtml). W1, W2, W3 and W4 with black solid arrows indicate the pathways of the four streams of the winter monsoon; I1 with green solid arrow and I2 with light green solid arrow indicate the pathways of the two streams of the Indian monsoon; and E1, E2 and E3 with blue solid arrows indicate the pathways of the three streams of the East Asian monsoon.

It should be pointed out that the precipitation isotope data at some GNIP stations are incomplete in the IAEA database. A critical analysis on the data quality should be carried out first, as done in many previous studies^8^,^43^. Based on such an analysis, 42 stations (28 stations in China and 14 stations in neighboring countries) within the GNIP were selected (Table 1 and Fig. 1). All the stations have isotopic records of more than two years with less than three monthly average data points missing. Monthly averaged values of D and ^18^O from each station were taken as the raw data. The recorded data of D or ^18^O at each station are regarded as a set of random variables. Given the independence of each isotope value, the precipitation isotope data are expected to follow the normal distribution. We used the data from the Hong Kong station and New Delhi station to test and validate this (supplementary information SI4). These two stations have isotopic records of 49 and 50 years, respectively. The variance of the isotope data for the record period is calculated and subsequently used in Eqn. 3 to compute the D and ^18^O entropy for each station.

### Data availability

All data used in the study are available from the GNIP Database. Accessible at: http://www.iaea.org/water.

## Additional Information

**How to cite this article**: Wang, T. *et al.* Entropy analysis of stable isotopes in precipitation: tracing the monsoon systems in China. *Sci. Rep.* **6**, 30389; doi: 10.1038/srep30389 (2016).

## Supplementary Material

Supplementary Dateset 1

Supplementary Information

## Figures and Tables

**Table 1:** Information about the 42 selected stations and values of the variance and entropy of D and ^18^O.

No.	Station	Latitude (decimal degrees)	Longitude (decimal degrees)	Start date	End date	D variance	D entropy	^18^O variance	^18^O entropy	Deviation#
1	Baotou	40.67	109.85	Jan-86	Dec-93	486.63	2.24	11.40	1.43	0.05
2	Changchun	43.9	125.22	Jan-99	Dec-01	270.11	2.11	15.79	1.50	0.19
3	Changsha	28.2	113.07	Jan-88	Dec-92	483.21	2.24	5.95	1.29	0.05
4	Chengdu	30.67	104.02	Jan-86	Dec-99	475.22	2.24	10.06	1.40	0.03
5	Fuzhou	26.08	119.28	Jan-85	Dec-92	233.22	2.08	4.33	1.22	0.02
6	Guangzhou	23.13	113.32	Jan-86	Dec-89	307.51	2.14	5.25	1.26	0.01
7	Guilin	25.07	110.08	Jan-83	Dec-90	524.49	2.26	7.17	1.33	0.03
8	Guiyang	26.58	106.72	Jan-88	Dec-92	838.08	2.36	9.91	1.40	0.06
9	Haikou	20.03	110.35	Jan-88	Dec-00	400.56	2.20	6.11	1.29	0.01
10	Haerbin	45.68	126.62	Jan-86	Dec-97	493.02	2.25	13.48	1.46	0.07
11	Hongkong	22.32	114.17	Jan-61	Dec-09	329.05	2.16	4.65	1.23	0.02
12	Khotan	37.13	79.93	Jan-88	Dec-92	4312.65	2.72	59.77	1.79	0.05
13	Kunming	25.02	102.68	Jan-86	Dec-03	616.64	2.29	13.71	1.47	0.04
14	Lanzhou	36.05	103.88	Jan-85	Dec-99	1473.57	2.48	33.27	1.66	0.03
15	Lhasa	29.7	91.13	Jan-86	Dec-92	1906.23	2.54	26.99	1.61	0.04
16	Liuzhou	24.35	109.4	Jan-88	Dec-92	387.31	2.19	6.43	1.30	0.00
17	Nanjing	32.18	118.18	Jan-87	Dec-92	311.84	2.15	4.18	1.21	0.03
18	Shijiazhuang	38.03	114.42	Jan-85	Dec-03	2686.79	2.08	44.22	1.27	0.06
19	Tianjin	39.1	117.17	Jan-88	Dec-01	226.91	2.19	5.49	1.32	0.02
20	Tsitsihar	47.38	123.92	Jan-88	Dec-92	375.88	2.61	7.06	1.72	0.02
21	Urumchi	43.78	87.62	Jan-86	Dec-03	339.34	2.49	4.38	1.65	0.02
22	Wuhan	30.62	114.13	Jan-86	Dec-98	1492.77	2.16	31.22	1.22	0.04
23	Xi’an	34.3	108.93	Jan-85	Dec-93	221.75	2.07	3.69	1.18	0.01
24	Yantai	37.53	121.4	Jan-86	Dec-91	114.50	1.93	3.03	1.14	0.08
25	Yinchuan	38.48	106.22	Jan-88	Dec-00	1682.41	2.51	26.94	1.61	0.02
26	Zhangye	38.93	100.43	Jan-86	Dec-03	1844.20	2.53	37.26	1.68	0.01
27	Zhengzhou	34.72	113.65	Jan-85	Dec-92	191.58	2.04	3.50	1.17	0.02
28	Zunyi	27.7	106.88	Jan-86	Dec-92	719.89	2.33	10.12	1.40	0.03
29	Bagdarin	54.47	113.58	Jan-96	Dec-00	2399.73	2.59	36.06	1.68	0.04
30	Barabinsk	55.33	78.37	Jan-96	Dec-00	1744.54	2.52	37.93	1.69	0.02
31	Cheongju	36.62	127.46	Jan-98	Dec-09	112.61	1.92	1.00	0.90	0.08
32	Hanoi	21.05	105.80	Jan-04	Dec-07	829.58	2.36	12.56	1.45	0.02
33	Hyderabad	17.45	78.47	Jan-97	Dec-01	780.28	2.35	14.03	1.47	0.00
34	Irkutsk	52.27	104.35	Jan-69	Dec-90	2089.70	2.56	30.97	1.64	0.04
35	Luang-Prabang	19.88	102.13	Jan-61	Dec-67	659.95	2.31	9.63	1.39	0.03
36	New Delhi	28.58	77.2	Jan-60	Dec-09	574.96	2.28	10.06	1.40	0.00
37	Pohang	36.03	129.38	Jan-61	Dec-76	127.24	1.95	0.97	0.89	0.10
38	Shillong	25.57	91.88	Jan-66	Dec-78	732.09	2.33	13.59	1.47	0.01
39	Terney	45	136.67	Jan-96	Dec-00	72.67	1.83	1.30	0.96	0.03
40	Tokyo	35.68	139.77	Jan-60	Dec-79	41.49	1.71	1.02	0.90	0.08
41	Ulaanbaatar	47.93	106.98	Jan-90	Dec-01	3611.30	2.68	58.27	1.78	0.03
42	Uttarkashi	30.73	78.45	Jan-04	Dec-06	992.28	2.40	13.64	1.47	0.04
	Maximum						2.72		1.79	
	Minimum						1.71		0.89	

^#^Deviation is the difference between the calculated and fitted entropy value.
