# Supplementary material for: Entropy analysis of stable isotopes in precipitation: tracing the monsoon systems in China
Source: Sci Rep. 2016 Aug 10;6:30389. doi: 10.1038/srep30389 (PMC4979205; doi:10.1038/srep30389)
Supplement: Supplementary Information [file srep30389-s2.pdf]

Supplementary Information

Title: Entropy analysis of stable isotopes in precipitation: tracing the monsoon systems in China

Tao Wang, Jiansheng Chen, Ling Li

SI1: Weighted mean (weighted by precipitation) δD and δ^{18}O isolines of the study area

Figs. S1 and S2 show results from the analysis of precipitation isotope data based on long-term weighted mean, which reveal less details of the links of the isotope with monsoon systems.

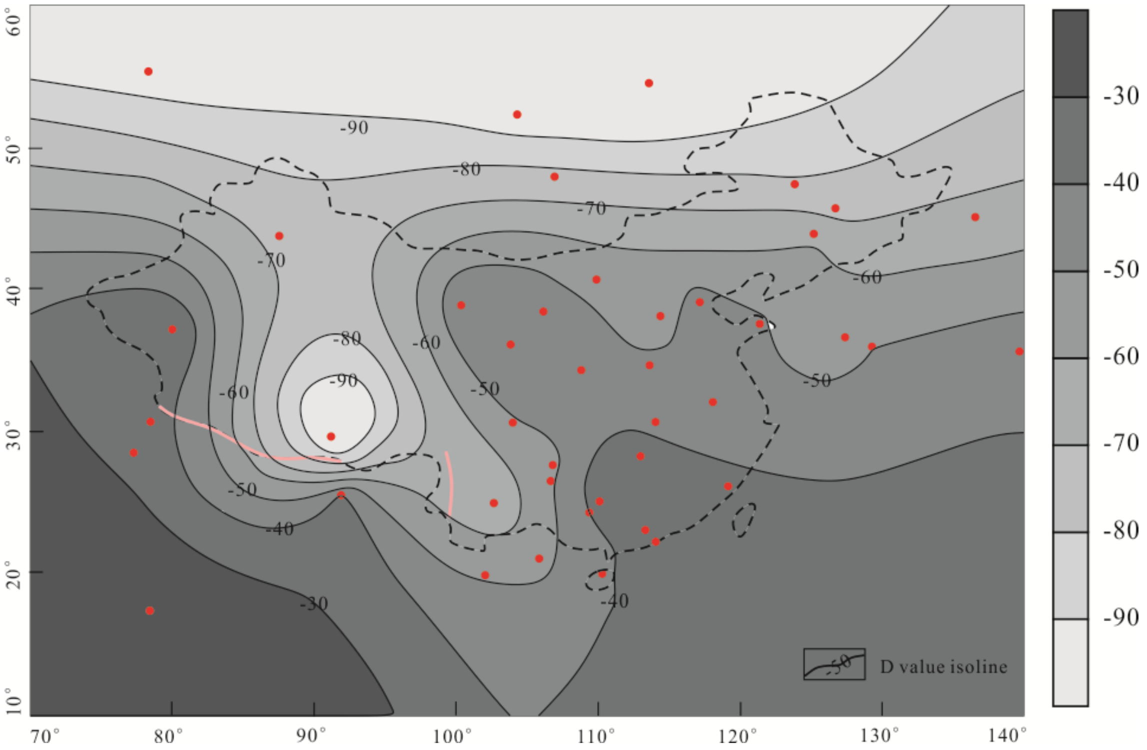

Figure S1 Weighted mean (weighted by precipitation) δD isolines of the study area, generated by Surfer® [12] from Golden Software, LLC (www.goldensoftware.com) and CorelDRAW X6 (http://down.52pk.com/xiazai/13513.shtml).

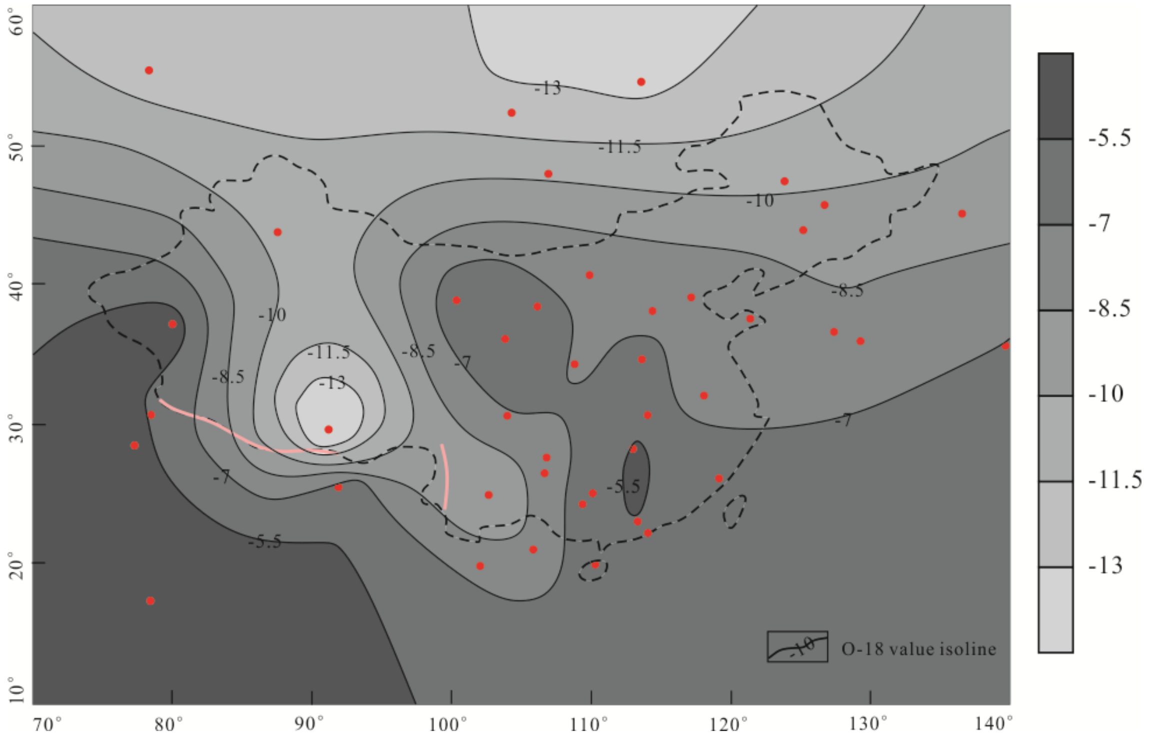

Figure S2 Weighted mean (weighted by precipitation) δ^{18}O isolines of the study area, generated by Surfer® [12] from Golden Software, LLC (www.goldensoftware.com) and CorelDRAW X6 (http://down.52pk.com/xiazai/13513.shtml).

SI2: Relation of D and ^{18}O entropy with annual precipitation average and annual temperature average

Figs. S3 and S4 show that there is no relation of the D and ^{18}O entropy with annual average precipitation or annual average temperature, indicating negligible effect of both factors on the variations of the precipitation isotope entropy.

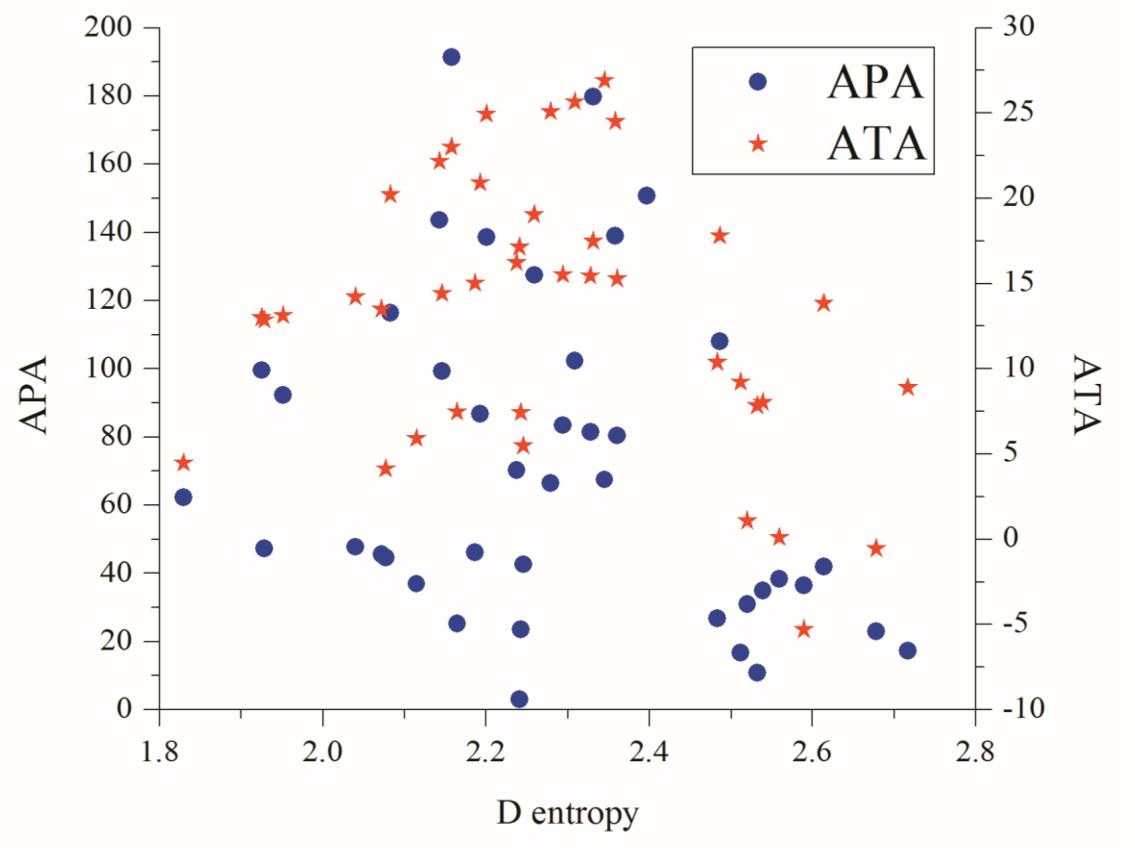

Figure S3 Variation of D entropy versus annual precipitation average (APA) and annual temperature average (ATA).

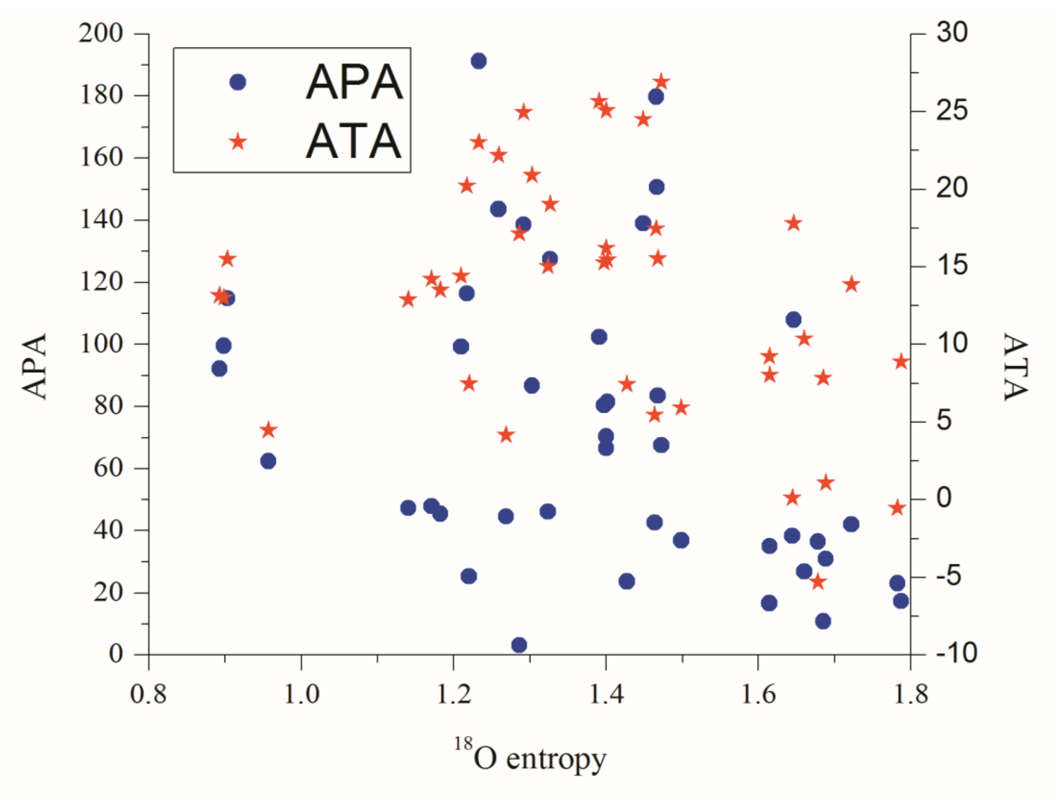

Figure S4 Variations of ^{18}O entropy versus annual precipitation average (APA) and annual temperature average (ATA).

S3: Analysis of deviation

Figs. S5 and S6 show that the deviation has no correlation with the longitude, latitude and monsoon pattern, annual precipitation average or annual temperature average. Fig. S7 shows that the deviation does not follow the normal distribution.

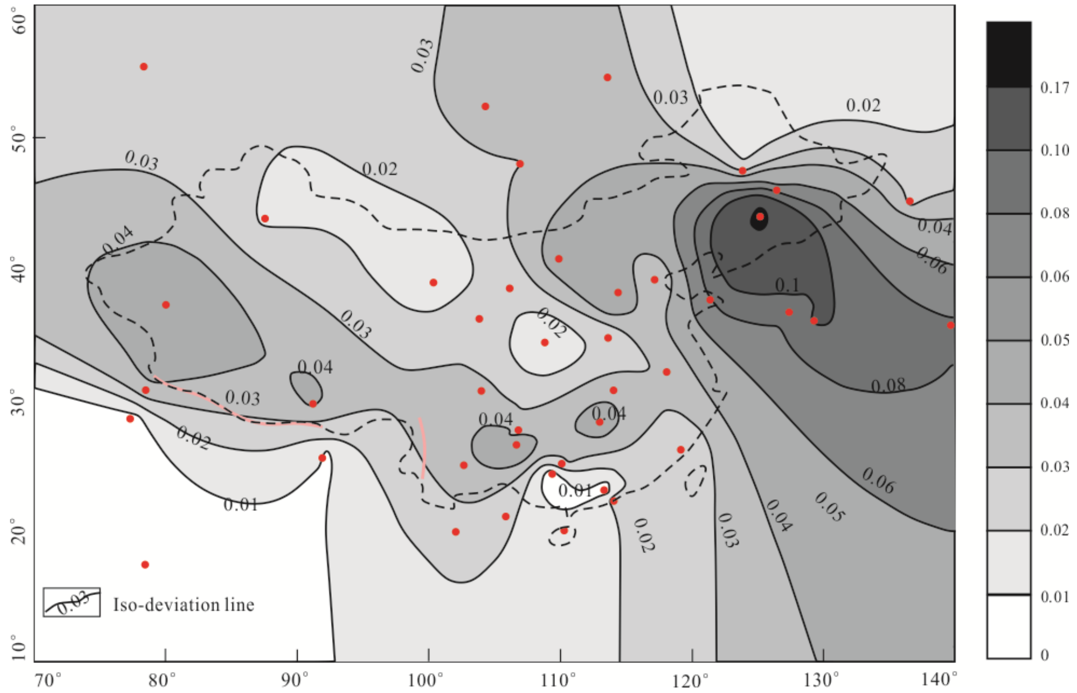

Figure S5 Variations of deviation with longitude, latitude and monsoon pattern, generated by Surfer® [12] from Golden Software, LLC (www.goldensoftware.com) and CorelDRAW X6 (http://down.52pk.com/xiazai/13513.shtml).

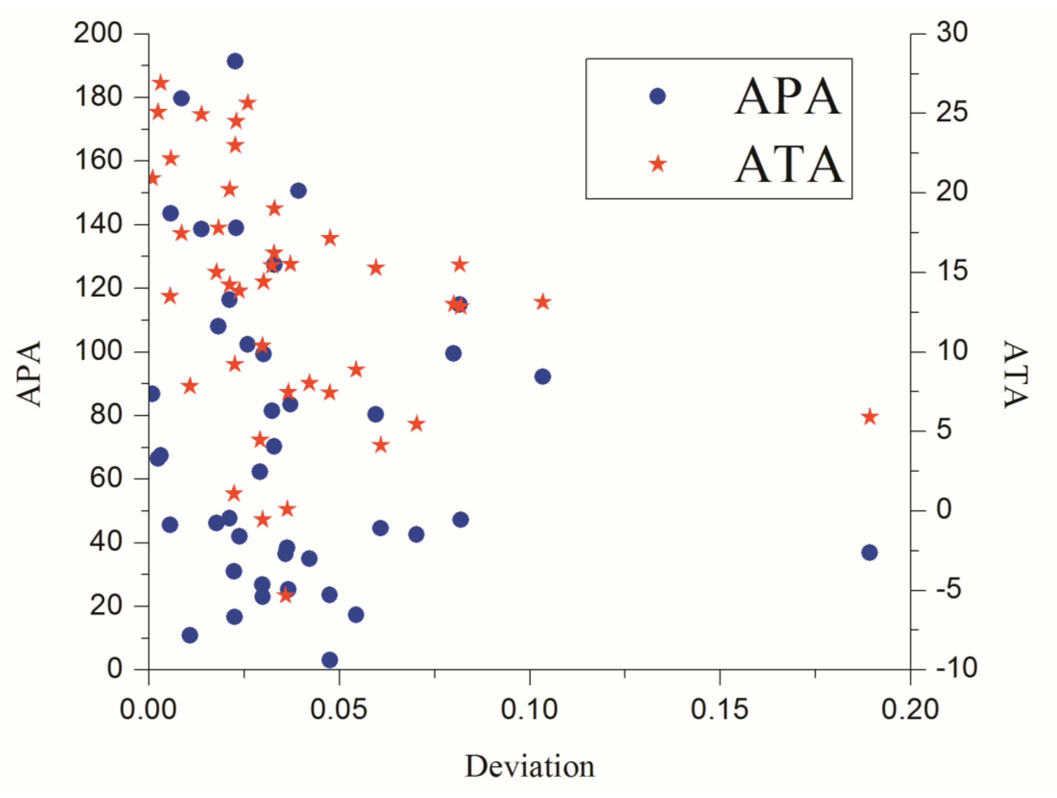

Figure S6 Variations of deviation versus annual precipitation average (APA) and annual temperature average (ATA).

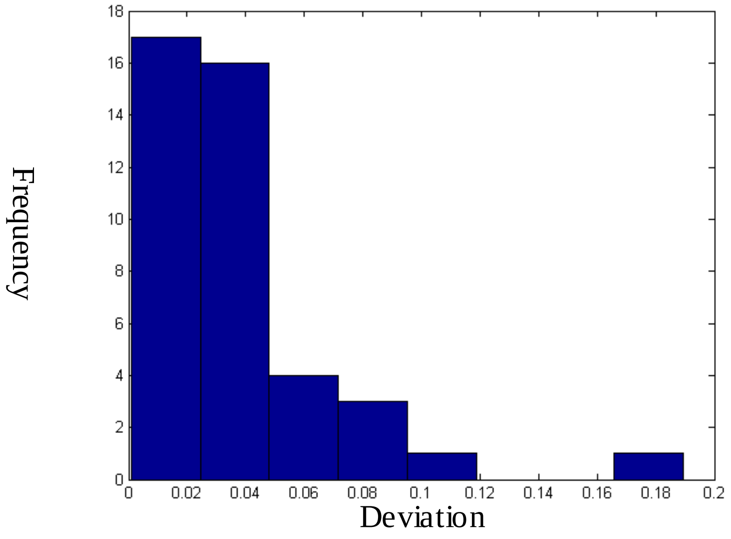

Figure S7 Frequency histogram of deviation.

SI4: The normal distribution of precipitation isotope data

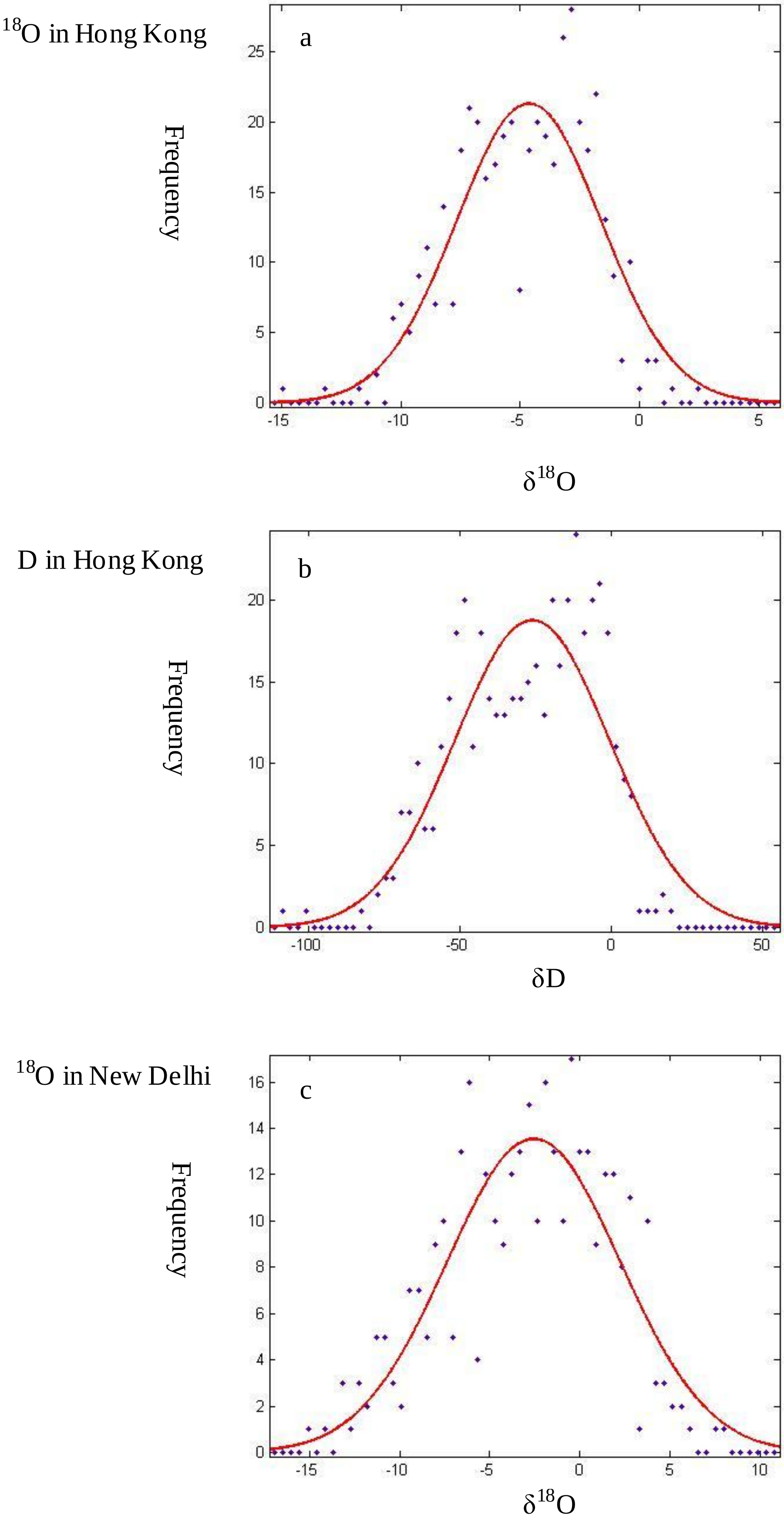

18O in Hong Kong
a
Frequency
0
5
10
15
20
25
-15
-10
-5
0
5
δ18O
D in Hong Kong
b
Frequency
0
5
10
15
20
-100
-50
0
50
δD
18O in New Delhi
c
Frequency
0
2
4
6
8
10
12
14
16
-15
-10
-5
0
5
10
δ18O

D in New Delhi

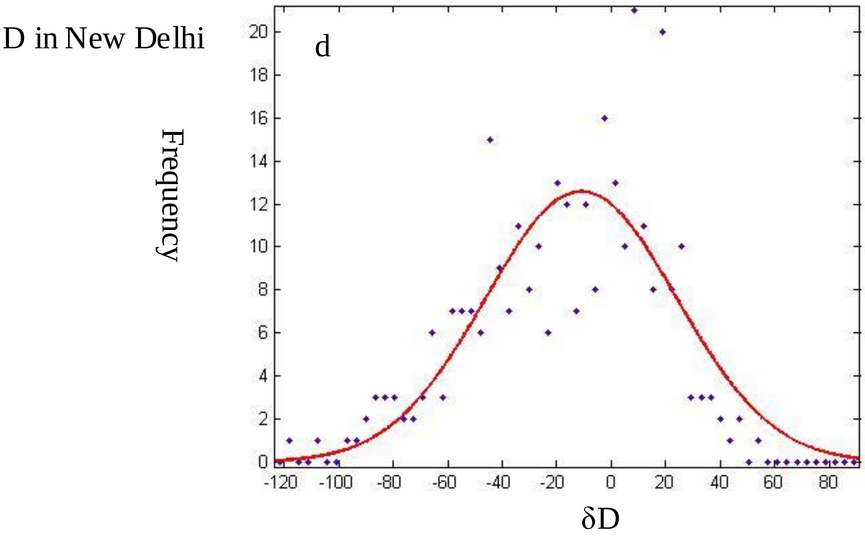

Figure S8 Fitted normal distribution curves of D and ^{18}O in precipitation based on data from the Hong Kong and New Delhi stations.

The fitted normal distribution curves of D and ^{18}O in precipitation in the Hong Kong and New Delhi stations are shown above. The fitting was done using the curve-fitting tool of MATLAB R2008a. Fig. S8a shows the fitting of ^{18}O in Hong Kong (R-Square = 0.8358); Fig. S8b shows the fitting of D in Hong Kong (R-Square = 0.8237); Fig. S8c shows the fitting of ^{18}O in New Delhi (R-Square = 0.8105); and Fig. S8d shows the fitting of D in New Delhi (R-Square =0.7185). Overall the results indicate that the precipitation isotope data follow the normal distribution reasonably well.
